# MicroRNA expression profile of chicken jejunum in different time points *Eimeria maxima* infection

**DOI:** 10.3389/fimmu.2023.1331532

**Published:** 2024-01-15

**Authors:** Endashaw Jebessa, Semiu Folaniyi Bello, Lijin Guo, Merga Daba Tuli, Olivier Hanotte, Qinghua Nie

**Affiliations:** ^1^ Department of Animal Genetics, Breeding and Reproduction, College of Animal Science, South China Agricultural University, Guangzhou, China; ^2^ Guangdong Provincial Key Lab of Agro-Animal Genomics and Molecular Breeding and Key Lab of Chicken Genetics, Breeding and Reproduction, Ministry of Agriculture, Guangzhou, China; ^3^ LiveGene-Centre for Tropical Livestock Genetics and Health (CTLGH), International Livestock Research Institute (ILRI), Addis Ababa, Ethiopia; ^4^ College of Pastoral Agriculture Science and Technology, Lanzhou University, Lanzhou, China; ^5^ School of Life Sciences, University of Nottingham, University Park, Nottingham, United Kingdom

**Keywords:** microRNA, expression profile, chicken jejunum, host immunity, *Eimeria maxima*

## Abstract

Coccidiosis stands as a protozoan disease of notable economic impact, characterized by an intracellular parasite that exerts substantial influence over poultry production. This invasion disrupts the integrity of the enteric mucosa, leading to the emergence of severe lesions and diminishing the efficiency of feed utilization in chickens. MicroRNA (miRNA) are short, non-coding RNA molecules with approximately 21–24 nucleotides long in size that play essential roles in various infectious diseases and inflammatory responses. However, the miRNA’s expression patterns and roles in the context of *Eimeria maxima* infection of chicken intestines remain unclear. miRNA sequencing was employed to assess the miRNA expression profile in chicken jejunum during *E. maxima* infection. In this study, we analyzed miRNA expression profiles related to the host’s immune response in the chicken jejunum during *E. maxima* infection. At 4 days infection and control (J4I versus J4C), 21 differentially expressed miRNAs in the jejunum were identified, comprising 9 upregulated and 12 downregulated miRNAs. Furthermore, in the jejunum, at 7 days infection and control (J7I versus J7C) groups, a total of 35 significantly differentially expressed miRNAs were observed, with 13 upregulated and 22 downregulated miRNAs. The regulatory networks were constructed between differentially expressed miRNA and mRNAs to offer insight into the interaction mechanisms between chickens and *E. maxima* coccidian infection. Furthermore, within the comparison group, we obtained 946, 897, and 281 GO terms that exhibited significant enrichment associated with host immunity in the following scenarios, J4I vs. J4C, J7I vs. J7C, and J4I vs. J7I, respectively. The KEGG pathway analysis indicated notable enrichment of differentially expressed miRNAs in the jejunum, particularly in J4I vs. J4C; enriched pathways included metabolic pathways, endocytosis, MAPK signaling pathway, regulation of actin cytoskeleton, and cytokine–cytokine receptor interaction. Moreover, in J7I vs. J7C, the KEGG pathway was significantly enriched, including metabolic pathways, protein processing in the endoplasmic reticulum, ubiquitin-mediated proteolysis, and FoxO signaling pathway. A comprehensive understanding of the host genetic basis of resistance with a combination of time-dependent infection to the *Eimeria* parasite is crucial for pinpointing resistance biomarkers for poultry production.

## 1 Introduction

Avian coccidiosis is a significant parasitic disease in chickens caused by the intracellular apicomplexan protozoa *Eimeria*, which specifically affects the intestinal tract, ultimately resulting in a severe and devastating disease (1). *Coccidiosis* is a typical chicken disease that affects different ages and breeds, and it causes significant morbidity and mortality in the global poultry industry (2). *Eimeria* parasite species have a high level of site specificity in the host, and each species invades intestinal epithelial cells in a distinct region of the gut, causing various subsequent levels of tissue damage and morbidity (3). Among seven recognized *Eimeria* species in chickens, *E. maxima*, a highly pathogenic parasite coccidian disease, primarily colonized the jejunum tract where it causes mucosal lesions and affects the metabolism and absorption of nutrients (4).

miRNAs are newly identified non-coding RNAs that can influence immune response, cell proliferation, differentiation, and apoptosis by regulating the expression of target mRNAs (5). They are tasked with regulating mRNA through degradation and fine-tuning protein levels (6). These miRNAs are involved in various cellular processes, including immune responses (innate and adaptive immune systems) and disease progression. Small RNA regulates cellular processes related to the host’s immune responses, including apoptosis, motility, and cell differentiation (7, 8). They play a vital role in many immune-related pathways, including the Toll-like receptor (TLR) signaling pathway, Wnt signaling pathway, and mitogen-activated protein kinase (MAPK) signaling pathway (9–11). They play pivotal regulatory roles in the interaction between host and parasitic pathogens by inhibiting target mRNAs at the post-transcriptional level (12, 13). miRNAs will regulate gene expression primarily by binding to the 3' untranslated regions of target mRNAs, which affect the translation process by inducing mRNA cleavage (14).

Infection by parasites can alter the expression of host miRNAs, potentially influencing both the parasite’s clearance and the infection’s progression (15). RNA silencing by the action of miRNAs plays a significant role in innate anti-parasitic and antibacterial defenses in animals (16). Regarding parasitic diseases like *Eimeria* infection in chickens’ intestines, miRNAs could modulate the expression of immune-related genes, affecting the outcome of the infection. As such, miRNA function analyses can provide invaluable insights into different aspects of the disease process and its effects. In addition, miRNAs, participating in the differentiation and proliferation of different cell populations in the intestine, can improve gut function (17, 18).

Comparing miRNA profile expressions between chicken breeds or populations with varying susceptibility to *Eimeria* infection could provide insights into the evolutionary aspects of host–parasite interactions. Indeed, miRNAs are central to the gene expression of host–parasite interaction, with the host cells miRNAs implicated in the elimination of the pathogen (19).

The miRNA high-sequence technology has offered a deeper exploration of miRNA involvement in poultry parasitic diseases (20, 21). The analysis of miRNA functions and their potential as biomarkers proves invaluable for examining various facets of parasitic diseases in chickens, including classification, diagnosis, and treatment. However, there is insufficient valuable data regarding the expression profiles of host miRNAs during various time points of *E. maxima* infection.

This study aimed to investigate host miRNA expression in the chicken jejunum at 4 and 7 days following infection with *E. maxima*, and the investigation utilized miRNA sequencing technology in combination with bioinformatics analysis. miRNA sequencing using high-throughput technology will enable a comprehensive analysis of miRNA expression patterns and their regulatory roles during coccidian infection in the chickens’ jejunum. This approach enhances the understanding of the infection’s molecular mechanisms and offers potential avenues for developing therapeutic strategies to combat the disease.

## 2 Materials and methods

### 2.1 Animals ethics

All the experimental procedures in this study complied with animal welfare protocols. All efforts were adopted to minimize animal suffering following relevant guidelines and regulations of the Institute Animal Care the Use Committee (IACUC) of the International Livestock Research Institute (ILRI) poultry research facility in Addis Ababa, Ethiopia. The protocol was approved by the ILRI, IACUC committee with reference number IACUC-RC2019-01.

### 2.2 Experimental design and sample collection

Fertilized eggs from the Ethiopian Horro chicken breed underwent incubation in an automated incubator at 37.5°C and relative humidity level of 78%. A total of 48 one-day-old Horro chickens were divided randomly into two groups, namely, the infected group (IG) and the control group (CG), each consisting of 24 chickens, with four replicates per group (n = 6). The chickens were kept in starter brooder units at the ILRI poultry facility in Addis Ababa, Ethiopia. They were subjected to a temperature-controlled environment following a standard protocol and had access to starter diet feed and water *ad libitum*. *Eimeria maxima* oocysts (supplied by Foshan Standard Bio-Tech Co., Ltd.) were administered to the challenge group of chickens via oral gavage at 21 days old, with each chicken receiving 2 ml containing 7×10^4^ sporulated oocysts. The same volume of distilled water was inoculated to each chicken in the control groups. To determine the impact of *E. maxima* infection on the chicken intestinal tract, 32 chickens were chosen from the infected and control groups.

In accordance with the life cycle of the *Eimeria* parasite within the chicken intestine, coccidia disease has an impact and leads to lesion formation at 4 and 7 days post-infection. At 4 and 7 days post-infection, eight chickens in two groups were humanely killed, respectively. The chicken was killed without pain, suffering, or distress, followed by death. Lesion scoring was performed according to the scoring technique of Reid and Johnson (22). Each selected chicken gastrointestinal tract (GIT) was dissected, and 200 mg of the jejunum tissue was collected immediately post-mortem from eight chickens in each group at 4 and 7 days post-infection. Subsequently, the tubes containing the collected samples were frozen and stored at −80°C until the extraction of total RNA.

### 2.3 RNA extraction and quality control

Total RNAs at 4 and 7 days post-infection of the chicken jejunum tissue were isolated from three individual samples, each from infection and control groups, using the RNeasy Min Elute Cleanup Kit (Qiagen, Hilden, Germany) according to the manufacturer guidelines and stored at −80°C. All RNA samples were briefly tested for RNA degradation and potential contamination on a 1.5% agarose gel electrophoresis. The RNA purity was tested using a 2000 NanoDrop (Thermo Fisher Scientific, USA), with all samples within the expected OD260/280 = 1.8–2.0 ratio, supporting optimal RNA purity. The RNA integrity was assessed using Bioanalyzer 2100 (Agilent, Palo Alto, CA, United States). All samples with RNA integrity numbers (RINs) ≥7 were used for miRNA sequencing at an Illumina Novoseq 6000 platform.

### 2.4 Small RNA library preparation and sequence analyzing

**Figure 1:**
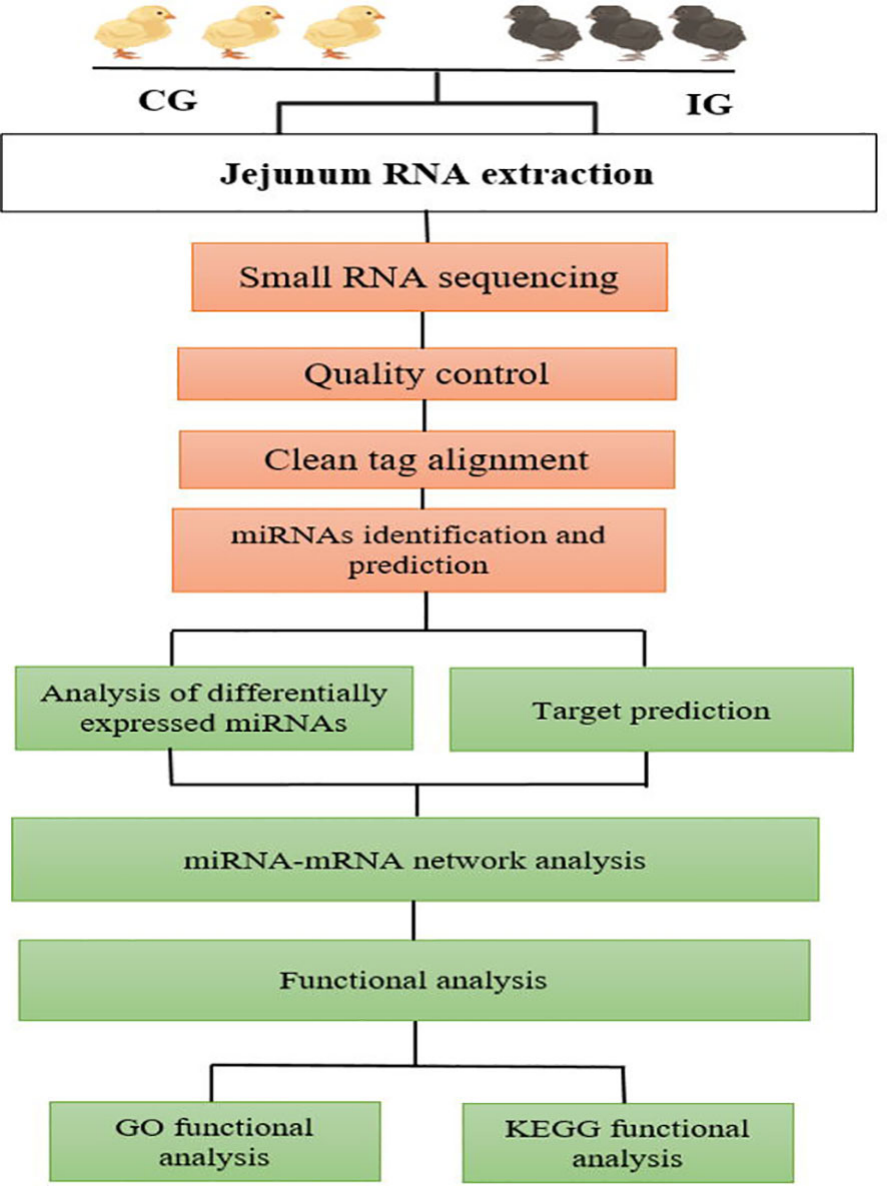
Workflow of data analysis and bioinformatics pipeline chart.

A total of 12 libraries were constructed from the chicken jejunum (4 and 7 days post-infection and control groups), with three replicates in each group. Approximately 2 μg of RNA of each sample was required for the miRNAs library preparation. According to the manufacturer’s recommendations, the small RNA libraries for Illumina sequencing were generated using the NEBnext Multiplex Small RNA Library Prep Set for Illumina (NEB, USA). The small RNA sequencing data analysis is outlined at **Figure 1**. The 3' and 5' adaptors were ligated to small RNA’s 3′ and 5′ ends, respectively. Subsequently, first-strand cDNA synthesis was accomplished by reverse-transcription reaction, followed by PCR amplification. The double-stranded cDNA library was generated through PCR enrichment. After purification and size selection, libraries with insertions between 18 and 40 bp were ready for sequencing on Illumina sequencing with SE50.

### 2.5 MiRNAs sequence data analysis

Raw fastq format quality control data were processed through custom Perl and Python scripts. Clean data were obtained by removing two-fifths reads containing poly-N with 5' adaptor contaminants, without 3' adaptor or insert tag, containing poly A or T or G or C, and low-quality reads from raw data. At the same time, the raw data Q20, Q30, and GC content were calculated. The small RNA tags were Bowtie mapped to the reference sequence without missing to analyze their distribution and expression (23). miRBase20.0 was used as a reference; modified software mirdeep2 (24) and small RNA-tools-cli were used to get the potential miRNA and draw the secondary structures. Specialized scripts were employed to obtain miRNA counts and to analyze the base bias at the initial position of identified miRNAs with specific lengths. Small RNA tags were aligned to databases such as Repeat Masker and Rfam, or similar data sources specific to the given species, to eliminate tags that originated from protein-coding genes, repetitive sequences, rRNA, tRNA, snRNA, and snoRNA. We integrated the miREvo (25) and mirdeep2 (24) software tools to predict novel miRNAs by examining the secondary structure, the Dicer cleavage site, and the minimum free energy of small RNA tags that were not annotated in the previous steps. To ensure that each distinct small RNA was assigned to only one annotation, the following priority rule was followed: known miRNA > rRNA > tRNA > snRNA > snoRNA > repeat > gene > NAT-siRNA > gene > novel miRNA > ta-siRNA.

### 2.6 Differentially expressed miRNAs analysis

The expression level of differentially expressed DEmiRNAs during *E. maxima* infection normalized the raw expression data by the transcript per million reads methods (26). Differential expression analysis of miRNAs of each group comparison sample was analyzed using the DESeq R package. The threshold of significantly different expression of miRNAs was set as *p*-value < 0.05 and |log2 (fold change)| >1.

### 2.7 Target gene prediction and functional analysis of miRNAs

The miRanda algorithm was employed to predict the target genes of differentially expressed miRNAs by scanning the 3' untranslated regions of the *Gallus gallus* reference genome. Gene ontology (GO) enrichment analysis was applied to the target gene candidates of differentially expressed miRNAs. Then, miRNA targets were subjected to GO enrichment analysis by calculating the *p*-value using R based on the hypergeometric distribution. Three terms in the GO database were mapped, including biological process (BP), cellular component (CC), and molecular function (MF). In addition, the pathway database within the KEGG (27) database was used to identify the pathways participated in by the DE miRNAs to understand the biological functions of the genes, from molecular-level information in the cell, the organism, and the ecosystem. KOBAS software was used to perform the statistical enrichment of candidate target genes in the KEGG pathway (28).

### 2.8 Statistical analysis

The Student’s t-test within the software GraphPad Prism 7.0 (http://www.graphpad.com) was used to determine statistical differences between the infection and control group, with a *p*-value of 0.05 considered statistically significant.

## 3 Results

### 3.1 Analyses of miRNAs sequencing data

A total of 12 small RNA libraries from 4 and 7 days post-infected, and control groups of jejunum were used for miRNA sequence analysis. In the group post-infection, miRNA libraries from 4 and 7 days post-infection yielded 43,014,913 and 40,768,698 raw reads, respectively. Reversely, in the control group, miRNA libraries from 4 and 7 days yielded 44,421,741 and 45,357,486 raw reads, respectively. After removing low-quality reads and masking adaptor sequences, over 11,071,815 clean reads were obtained from each 12-miRNAs library (**Table 1**). The percentage of total mapped clean reads for each sRNA library was 80%–91.38% aligned with the chicken reference genome (*Galgal* GRCg6a). The GC content of 12 sRNA sample libraries was between 49.6% and 53.83%, and Q30 percentages were >96.79% (**Table 1**). The length of sRNA typically ranges from 18 to 40 nucleotide (nt), but the most abundant size sRNA in infected and control groups of jejunum was 21–24 nt.

**Table 1:** Statistical analysis of miRNAs sequence of the miRNA libraries.

Sample name	Raw reads	Clean reads	Total mapping	Aligned %	Q30%	GC %
J4I-1	13,502,144	13,179,948	12,044,402	91.38	97.15	49.6
J4I-2	15,715,509	15,326,443	13,266,589	86.56	97.37	50.64
J4I-3	13,797,260	13,386,634	11,580,680	86.51	97.5	50.23
J4C-1	13,487,228	12,958,779	10,411,858	80.34	97.32	51.81
J4C-2	17,453,183	15,871,777	12,992,637	81.86	97.18	51.98
J4C-3	13,481,330	12,645,934	10,418,985	82.39	96.79	53.83
J7I-1	14,644,801	14,054,167	11,251,282	80.06	96.99	51.28
J7I-2	12,963,279	11,071,815	8,868,797	80.10	96.81	51.96
J7I-3	13,160,618	12,645,170	10,727,658	84.84	97.34	50.88
J7C-1	17,424,980	17,027,330	15,214,441	89.35	97.51	50.17
J7C-2	14,771,888	14,443,947	12,595,136	87.20	97.55	50.93
J7C-3	13,160,618	14,454,431	13,166,743	91.09	97.48	50.32

Known conserved miRNAs were identified using miRBase (http://www.mirbase.org/). The hairpin structure of novel miRNA precursors was predicted using the miREvo and mirdeep2 to explore secondary structures, Dicer cleavage sites, and free energies of a certain length of sRNA tags. Thus, the present study identified mature miRNAs, 762 known and 216 novel mature miRNAs corresponding to 665 and 216 precursors, respectively, with a BLAST search against the miRBase (**Table 2**). Mature, known, and novel miRNAs were shared abundantly in 12 miRNA libraries. The most 10 abundant known miRNAs in each sample were gga-miR-143-3p, gga-miR-21-5p, gga-miR-425-5p, gga-let-7i, gga-miR-215-5p, gga-miR-30d, gga-let-7g-5p, gga-miR-148a-3p, gga-miR-194, and gga-let-7a-5p, and four novel miRNAs, novel_3, novel_1, novel_72, and novel_2 (**Supplementary Table S1**).

**Table 2:** The known and novel miRNAs mapped in the chicken genome.

Known miRNAs					Novel miRNAs				
Types	Mapped mature	Mapped hairpin	Mapped unique sRNA	Mapped Total sRNA	Mapped mature	Mapped star	Mapped hairpin	Mapped unique sRNA	Mapped Total sRNA
**Total**	762	655	38,862	66,589,293	216	74	216	2,193	30,414
**J4I-1**	522	468	3,718	7,593,257	96	13	107	182	2,847
**J4I-2**	529	473	3,611	7,319,662	98	16	118	216	3,323
**J4I-3**	504	464	3,502	6,489,518	107	10	123	220	3,593
**J4C-1**	469	440	3,272	3,539,744	105	20	118	266	1,353
**J4C-2**	462	435	2,960	3,739,379	97	10	112	188	2,089
**J4C-3**	419	387	2,542	2,496,340	71	9	77	120	1,276
**J7I-1**	498	452	3,351	5,703,765	95	9	108	189	4,957
**J7I-2**	427	396	2,533	2,3811,52	69	7	77	108	981
**J7I-3**	482	437	3,347	5,879,431	86	13	99	167	2,177
**J7C-1**	500	448	3,477	8,342,038	111	16	128	214	3,347
**J7C-2**	477	428	3,235	6,077,214	81	8	93	144	1,770
**J7C-3**	488	448	3,314	7,027,793	92	7	103	179	2,701

### 3.2 Differentially expressed miRNA in the jejunum during *Eimeria maxima* infection

miRNAs have the possibility to serve as biomarkers for diseases. By analyzing miRNA expression profiles in response to *Eimeria* infection, the study can identify specific miRNAs that were up- or downregulated. These miRNAs can then be evaluated as potential biomarkers for disease diagnosis, progression monitoring, and treatment efficacy assessment.

**Figure 2:**
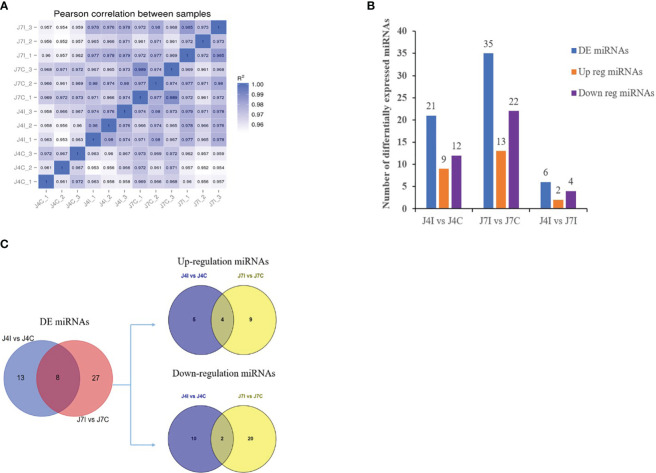
The differential expression miRNAs analysis of chicken jejunum between infected and control groups. **(A)** The Pearson correlation coefficients analysis between different samples, associated with the expression level of miRNAs. **(B)** The number of differentially expressed miRNAs between the infected and control comparison groups. **(C)** The Venn diagrams of the differentially expressed miRNAs between infected and control comparison groups.

Differential expressed analysis was used to determine differentially expressed miRNAs with *p*-value<0.05 and |Log2FC|>1. In the gene expression patterns of miRNAs in different samples, Pearson correlation coefficients were used to assess the gene expression levels. The correlation coefficients ranged from 0.952 for J7I-2 versus J4C-2 to 0.989 for J7C-1 versus J7C-3 (**Figure 2A**). A total of 62 unique chicken encoded miRNAs were significantly (*p<*0.05) differentially expressed between infected and control group samples at 4 and 7 days with *E. maxima* coccidian, including three novel miRNAs gga-novel_199 in J4I vs. J4C, gga-novel_110 in J7I vs. J7C, and gga-novel_2 in J4I vs. J7I (**Figure 2B**). A total of 40 differentially expressed miRNAs were identified in the unique sample, including 13 from J4I vs. J4C and 27 from J7I vs. J7C. In another way, eight differential miRNAs were shared in J4I and J7I sample groups, including gga-miR-212-5p, gga-miR-2184a-5p, gga-miR-132c-3p, gga-miR-27b-3p, gga-miR-1729-5p, gga-miR-1388b-3p, gga-miR-1388a-5p, and gga-miR-204 (**Figure 2C**).

### 3.3 Cluster and volcano plot analysis of the differences between miRNAs expressions

**Figure 3:**
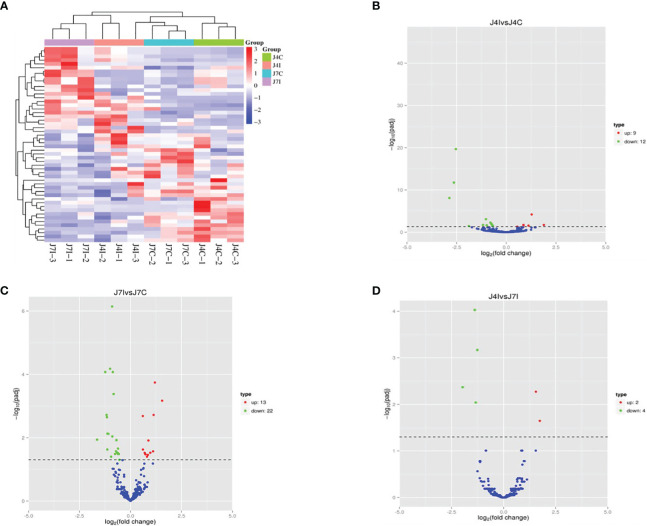
Differentially expressed miRNA cluster heat map and volcano plot analysis. **(A)** Heat map analysis of each sample of miRNAs with red indicated high expression of miRNAs and blue indicated low expression of miRNAs. **(B)** Volcano J4I vs. J4C, **(C)** volcano J7I vs. J7C, and **(D)** volcano J4I vs. J7I. The horizontal axis represents the log2 fold change of miRNA expression in different experimental groups.

miRNAs possess the capability to function as disease biomarkers. Through the examination of miRNA expression patterns in response to *Eimeria* infection, researchers can pinpoint particular miRNAs that exhibit up- or down-regulation. These miRNAs can then be evaluated as potential biomarkers for disease diagnosis, progression monitoring, and treatment efficacy assessment. Cluster heat map and volcano plot analysis were used for differentially expressed miRNA under different experimental conditions, using the threshold *p* < 0.05, and (|Log2FC>1|). The heat map was used to analyze differentially expressed miRNA patterns in infected and control samples at 4 and 7 days post-infection with *E. maxima* (*p* < 0.05) (**Figure 3A**). A volcano plot revealed the overall distribution of different miRNA expressions; 21, 35, and 6 significantly (*p* < 0.05) differentially expressed miRNAs were discovered in J4I vs. J4C, J7I vs. J7C, and J4I vs. J7I comparison groups, respectively. Compared with J4C, 21 differentially expressed miRNAs were detected in J4I, including 9 upregulated and 12 downregulated miRNAs (**Figure 3B**). Furthermore, 35 significantly (*p*<0.05) differential expressed miRNAs were identified in J7I (13 upregulated and 22 downregulated miRNAs) compared with J7C (**Figure 3C**). Similarly, six differentially expressed miRNAs were observed between J4I and J7I, including two upregulated and four downregulated miRNAs (**Figure 3D**).

### 3.4 Differential expressed miRNA–target gene network analysis

Small RNAs can control gene expression by targeting the 3' UTR of mRNA, leading to the inhibition of their respective targets by suppressing translation. Therefore, miRDB was employed to predict target genes for differentially expressed miRNAs, aiming to unravel the potential functions and mechanisms of functional networking during *E. maxima* infection in the chicken jejunum.

**Figure 4:**
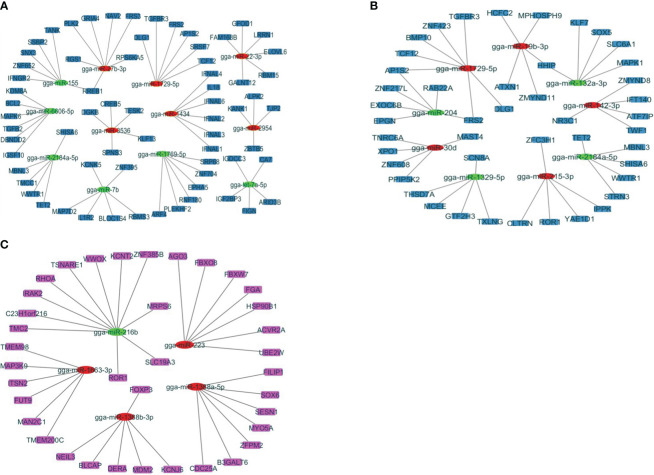
The network analysis of DEmiRNAs with the target genes involved in the immune response to *Eimeira maxima* infection of jejunum, predicted by miRDB. **(A)** J4I vs. J4C, **(B)** J7I vs. J7C, **(C)** J4I vs. J7I. The node used for representing the different miRNAs and target genes, which are connected by edges (negative interaction between miRNA to target gene). The green ellipses are upregulated miRNAs, the red ellipses are downregulated miRNAs, and the color rectangle nodes represent target genes.

Studying these miRNA–gene interactions can help to understand how the parasite evades or manipulates the host’s immune defenses. To investigate the regulatory functions of miRNAs, the study has established negative interactions between miRNAs and immune-related target genes that were differentially expressed at J4I and J7I, using Cytoscape 3.10 (**Figure 4**). In the J4I vs. J4C contrast group, 68 genes were possibly regulated by 12 differential expression miRNAs, including six upregulated and six downregulated miRNAs (**Figure 4A**). Among these target genes, the *RREB1* target gene was regulated by up- and downregulated miRNAs, gga-miR-155 and gga-miR-27b-3p, respectively (**Figure 4A**). At J7I vs. J7C (**Figure 4B**), 48 different genes were possibly regulated by nine differentially expressed miRNAs. Among these target genes, four upregulated miRNAs regulated 21 genes, and 27 were regulated by five downregulated miRNAs. Target genes, *FRS2* and *AP1S1*, were regulated by gga-miR-204 (up-regulated) and gga-miR-1729-5p (downregulated), while *HHIP* target genes were regulated by gga-miR-132a-3p (upregulated) and gga-miR-19b-3p (downregulated) (**Figure 4B**).

Contrarily, between J4I and J7I sample groups, 37 target genes were possibly regulated by five differentially expressed miRNAs. From these differentially expressed miRNA–mRNA negatively correlated network interactions, 11 target genes were regulated by one upregulated miRNA (gga-miR-216b), while 26 target genes were regulated by four downregulated miRNAs, including gga-miR-1388b-3p, gga-miR-223, gga-miR-1663-3p, and gga-miR-1388a-5p (**Figure 4C**).

### 3.5 Functional enrichment analysis of target genes of differentially expressed miRNAs

Candidate target genes of differentially expressed miRNAs were predicted using RNA Hyprid and miRanda software. The differentially expressed miRNA target genes 901, 1713, and 844 were predicted in J4I vs. J4C, J7I vs. J7C, and J4I vs. J7I comparison groups, respectively. To better understand the function of differentially expressed miRNAs, GO and KEGG pathway enrichment on the putative target genes was done. The GO terms include biological processes (BP), cellular components (CC), and molecular functions (MF). Compared with control groups, 946, 897, and 281 significantly enriched GO terms (*p*<0.05) related to immunity and inflammation were identified from J4I, J7I, and between J4I and J7I, respectively.

**Figure 5:**
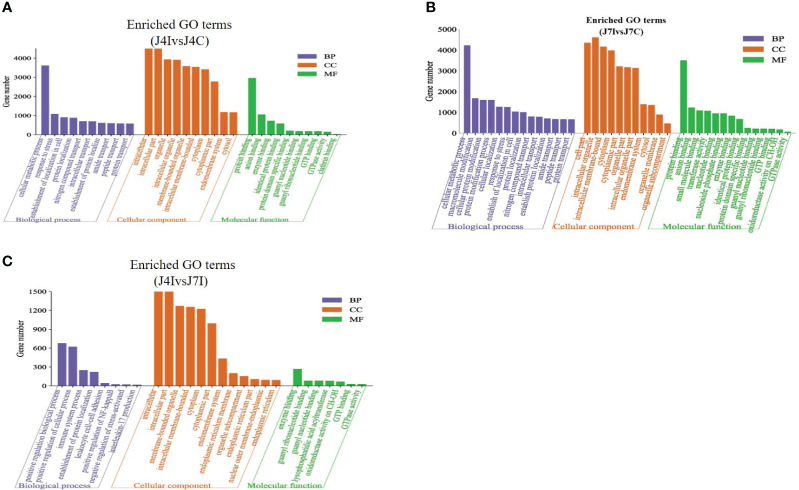
The GO enrichment analysis for target gene of differentially expressed miRNAs in chicken jejunum during *Eimeria maxima* infection. The significantly enriched (*p*<0.05) GO terms of biological process, cellular component and molecular function at **(A)** J4I vs. J4C sample group, **(B)** J7I vs. J7C sample group, and **(C)** J4I vs. J7I sample group. The X-axis indicates GO terms of biological process, cellular component, and molecular function, and the Y-axis the number of target genes.

Some GO enrichment functions were shared between two comparison groups, namely, J4I vs. J4C and J7I vs. J7C. These functions include cellular metabolic processes, peptide transport, amide transport, establishment of cellular localization, protein transport in biological processes (BP); cytoplasm, cytoplasmic part, intracellular part, membrane-bounded organelle, cell part, and intracellular membrane-bounded organelle in cellular components (CC); and identical protein binding, GTP binding, enzyme binding, GTPase activity, guanyl nucleotide binding, and guanyl ribonucleotide binding in molecular functions (MF) (**Figures 5A, B**). Furthermore, between J4I and J7I, GO term analysis was mostly involved in the positive regulation of NF-kappaB transcription factor activity, positive regulation of biological process, positive regulation of cellular process, immune system process, establishment of protein localization, leukocyte cell–cell adhesion, interleukin-17 production, negative regulation of stress-activated MAPK cascade, and lysophospholipid acyltransferase activity (**Figure 5C**).

### 3.6 KEGG pathway analysis

**Figure 6:**
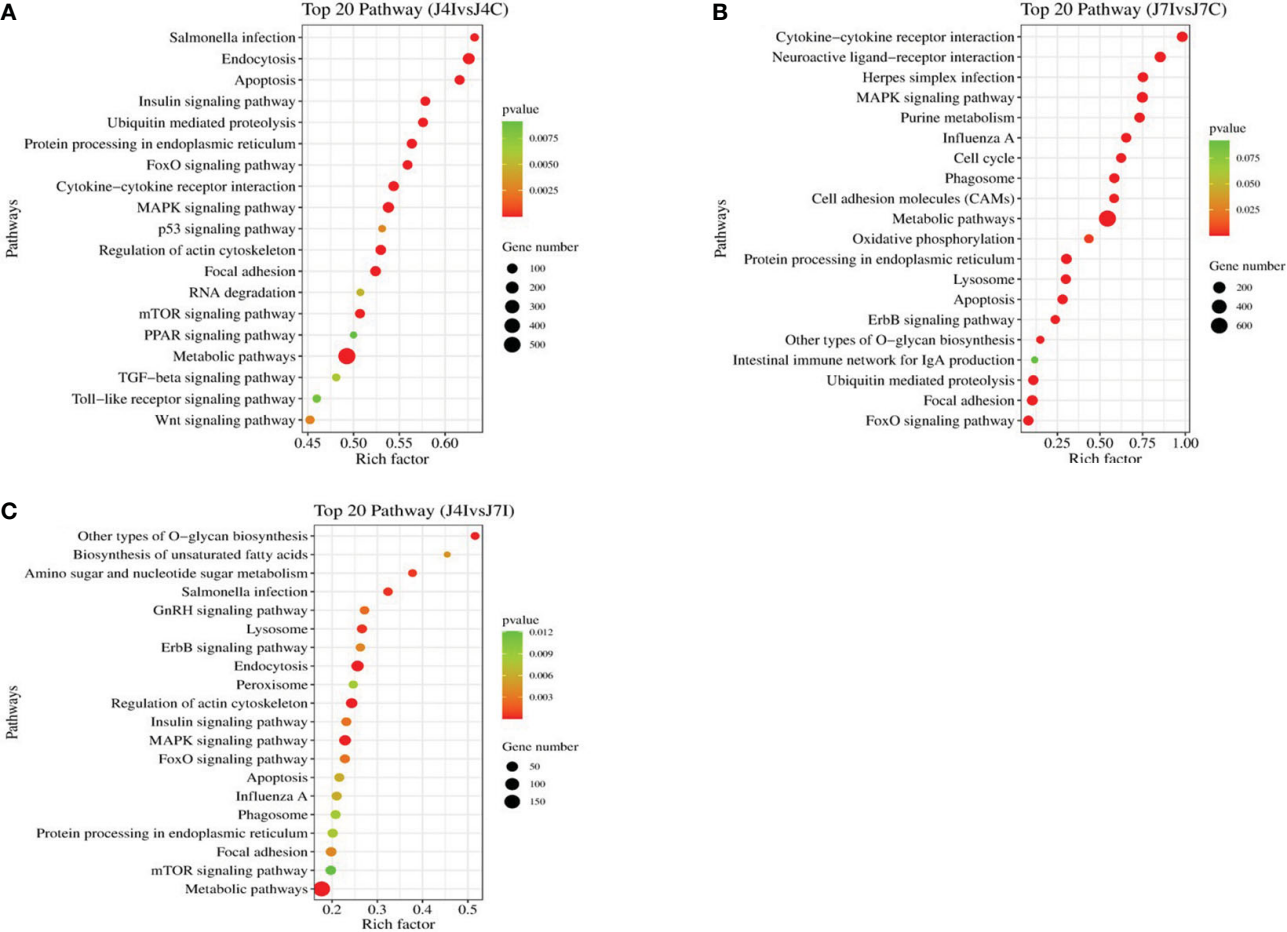
The histogram of KEGG pathway analysis for target genes of differentially expressed miRNAs. Top 20 KEGG pathway of differentially expressed miRNAs at **(A)** J4I vs. J4C, **(B)** J7I vs. J7C, and **(C)** J4I vs. J7I. The X-axis indicates the ratio of target genes of differential expressed miRNAs enriched pathway, and the Y-axis the KEGG pathway description.

Furthermore, the KEGG pathway in differentially expressed miRNAs target genes with *p*<0.05 was analyzed. A total of 153 and 152 KEGG pathways were identified in the comparisons of J4I vs. J4C and J7I vs. J7C, respectively. Additionally, 132 KEGG pathways were found differentially expressed between the J4I and J7I sample groups. The top 20 pathways of KEGG analysis of differentially expressed miRNA target genes were described in comparison groups with *p*-value <0.05 (**Figure 6**). From J4I vs. J4C, metabolic pathways, endocytosis, MAPK signaling pathway, apoptosis, regulation of actin cytoskeleton, and cytokine–cytokine receptor interaction were the main pathways significantly enriched (*p*<0.05) (**Figure 6A**). At J7I vs. J7C, main KEGG pathways, metabolic pathways, protein processing in the endoplasmic reticulum, cytokine–cytokine receptor interaction, lysosome, apoptosis, ubiquitin-mediated proteolysis, and FoxO signaling pathway were significantly enriched (*p*<0.05) (**Figure 6B**). Moreover, in J4I vs. J7I group samples, pathways, MAPK signaling pathway, regulation of actin cytoskeleton, metabolic pathways, and other types of O-glycan biosynthesis were significantly enriched (**Figure 6C**).

## 4 Discussion

*Eimeria* parasite coccidian causes yearly huge economic losses in poultry production (29, 30). Coccidiosis challenge caused significant adverse effects on performance traits, intestinal morphology, and hematological variables (31). *Eimeria* coccidiosis is a serious intestinal disease in different ages and breeds of chickens, compromising productivity and animal welfare (2). Hence, developing a novel plan for alleviating this disease should focus on the interaction between the host and *Eimeria* parasite species in chicken tissues. Combining the miRNA expression profile with transcriptome pathway functional analysis is vital to studying miRNA regulatory mechanisms during *Eimeria* parasite infection. Small RNA is an important post-transcriptional regulatory in various biological and molecular processes, including infectious diseases (12, 13). The *Eimeria* parasite species primarily affects chickens between 3 and 18 weeks of age, although it is worth remarking that significantly higher infection and mortality rates are observed among young chicks (32, 33). The study has reported that tributyrin supplement potentially improves the performance of broiler chickens during coccidiosis (34) (Hansen Victoria, et al., 2021). However, chicken breeds have shown different susceptibility levels to *Eimeria* species interims of gene transcriptome expression. We used the *E. maxima* coccidian infection of an early chicken age to investigate the miRNA profile expression in the jejunum. The jejunum tissue in chicken infected with *E. maxima* was collected 4 and 7 days post-infection to perform further experimental analysis. The differential expression profile of small RNAs, with transcriptome pathway functional enrichment, played a pivotal role in exploring the regulatory mechanisms of miRNAs during *Eimeria* coccidian infection in the chicken intestinal tract. We identified 978 differential miRNAs compared to the control group, in the infected samples, comprising 762 known miRNAs and 216 novel miRNAs. This analysis was conducted at both 4 and 7 days post-infection.

When comparing significantly differentially expressed miRNAs, we observed 9 upregulated and 12 downregulated miRNAs at 4 days post-infection. In contrast, at 7 days post-infection, 13 up-regulated and 22 down-regulated miRNAs were identified, many of which were related to the host’s immune response to coccidiosis. Interestingly, among upregulated differential expressed miRNAs, gga-miR-204, gga-miR-212-5p, gga-miR-2184a-5p, and gga-miR-132c-3p were shared at two contrast groups (J4I vs. J4C and J7I vs. J7C). Differentially expressed miRNAs will likely have significant roles in the chicken jejunum’s response to *E. maxima*. Recent studies have revealed the involvement of miRNAs, specifically gga-miR-204 and gga-miR-132c-5p, in maintaining intestinal mucosal integrity and regulating inflammatory responses (35, 36). miRNA expression of infected Ross 308 broilers with *E. maxima* using RNA sequencing, and gga-miR-144-3p, gga-miRA-122-5p, and gga-miR-205b were confirmed by qRT-PCR, which were involved during avian coccidiosis (37).

The regulatory networks miRNs–mRNA showed that *FRS2* and *AP1S2* target genes interacted with gga-miR-204 and gga-miR-1729-5p, while *HHIP* target gene is regulated by two miRNAs, gga-miR-19b-3p and gga-miR-132a-3p, at 7 days post-infection. Furthermore, gga-miR-155 and gga-miR-27b-3p were regulated by *RREB1* target genes at 4 days post-infection. The results suggest that the expression of miRNAs were altered in response to *E. maxima* infection and that they modulate the expression of their target genes during jejunum coccidian infection. The networking mechanisms of miRNAs and their target genes may contribute to the immune response by negatively regulating the production of inflammatory cytokines, thereby inhibiting the invasion of *Eimeria* parasites (38). Differentially expressed miRNA and predicted target genes regulatory network functional analysis plays a crucial role in the host response against *Eimeria* parasite infection (39). Several miRNAs, including miR-214b-3p, miR-200b-3p, and miR-92-3p, have been linked to host immunity and disease processes in *Eimeria tenella* infection (40). Additionally, miR-223 has been associated with gut inflammation and the disease infection process (41), while gga-miR-30a-5p and gga-miR-155 have been implicated in bursal disease during infection (42). Differentially expressed miRNAs and their target genes are known to have a specific role in regulating tumor formation including cell proliferation and apoptosis. Thus, gga-miR-155 was involved in increasing the proliferation, invasiveness, and reduced apoptosis by targeting *RORA* (43). Moreover, gga-miR-155 target sites in env transcripts significantly decreased levels of env transcripts abundance in MSB1 and CEF cells (44). The study revealed that the development and progression of inflammatory disease are very complex and are regulated by a molecular network involving multiple miRNAs and their target genes (45). Understanding miRNA–mRNA interactions during coccidian infections from this study can aid in developing therapies. Researchers can modulate the host’s immune response by targeting specific miRNAs or their target genes to enhance protection against the *Eimeria* parasites.

Furthermore, in this study, we investigated the potential target genes of differentially expressed miRNAs and conducted a screening of various GO terms associated with host immune enhancement. This analysis aimed to assess the functions of genes enriched in the jejunum during *E. maxima* infection. The main finding of GO term functional enriched revealed that the target gene of the differential expressed miRNAs at 4 and 7 days post-infections were involved in negative regulation of stress-activated MAPK cascade, positive regulation of biological process, interleukin-17 production, positive regulation of NF-kappaB transcription factor activity, immune system process, and positive regulation of cellular process. A previous study has reported that the MAPK signaling pathway plays a vital role for miRNAs in regulating chicken immune response and a mechanism of alleviating host inflammation during necrotic enteritis infection (46). Furthermore, the KEGG pathway analysis for target genes of differentially expressed miRNAs was identified at 4 and 7 days post-infection with coccidiosis infection. The immune-associated KEGG pathways in the top 20 enriched KEGG pathways included MAPK signaling pathway, cytokine–cytokine receptor interaction, metabolic pathways, endocytosis, Herpes simplex infection, and protein processing in the endoplasmic reticulum and mTOR signaling pathway, suggesting that the miRNAs play important roles in the immune response to *Eimeria* coccidiosis. The use of GO and KEGG enrichment analyses provided further gain insights into the potential impact of the infection on the host’s cellular and molecular functions.

By conducting target gene functional enrichment analysis on the differentially expressed microRNAs (miRNAs), we gain insights into the potential functional contributions of these miRNAs in coccidian-related conditions.

## 5 Conclusion

The present study has determined the miRNA expression profile in the jejunum of chicken infected with *E. maxima* at 4 and 7 days post-infection. It identified differentially expressed miRNAs and their target gene related to the immune response in chickens during infection. Specific miRNAs might correlate with the severity of *Eimeria* infection. By examining the expression patterns of these miRNAs in infected chickens, researchers can potentially predict the course of the disease.

GO and KEGG enrichment analysis revealed that *E. maxima* infection of chicken jejunum might modulate the host biological process through differential miRNA with their target genes. The host immune-associated pathways and GO terms, MAPK signaling pathway, cytokine–cytokine receptor interaction, negative regulation of stress-activated MAPK cascade, positive regulation of the biological process, and interleukin-17 production play roles during *E. maxim* infection. miRNA function analyses can significantly enhance the consideration of the complex interactions between *Eimeria* parasites and their chicken hosts during infection. They can provide valuable information for developing diagnostic tools, therapies, and strategies to manage and control parasitic diseases in poultry.

## Data availability statement

The data presented in the study are deposited in the NCBI SRA repository, accession number PRJNA1044851.

## Ethics statement

The animal study was approved by Institute Animal Care the Use Committee (IACUC), in International Livestock Research Institute in Addis Ababa, Ethiopia. Committee number with the reference IACUC-RC2019-01. The study was conducted in accordance with the local legislation and institutional requirements.

## Author contributions

EJ: Data curation, Formal Analysis, Investigation, Methodology, Writing – original draft. SB: Writing – review & editing. LG: Methodology, Writing – review & editing. MT: Writing – review & editing. OH: Conceptualization, Methodology, Resources, Supervision, Writing – review & editing. QN: Conceptualization, Methodology, Project administration, Resources, Supervision, Writing – review & editing.
